# Patterns of care for older patients with stage IV non‐small cell lung cancer in the immunotherapy era

**DOI:** 10.1002/cam4.2854

**Published:** 2020-01-27

**Authors:** Kenneth L. Kehl, Michael J. Hassett, Deborah Schrag

**Affiliations:** ^1^ Division of Population Sciences Dana‐Farber Cancer Institute and Harvard Medical School Boston MA USA

**Keywords:** geriatrics, health services research, immunotherapy, lung cancer, patterns of care, SEER‐Medicare

## Abstract

**Background:**

Historically, older patients with advanced lung cancer have often received no systemic treatment. Immunotherapy has improved outcomes in clinical trials, but its dissemination and implementation at the population level is not well‐understood.

**Methods:**

A retrospective cohort study of patients with stage IV non‐small cell lung cancer (NSCLC) diagnosed age 66 or older from 2012 to 2015 was conducted using SEER‐Medicare. Treatment patterns within one year of diagnosis were ascertained. Outcomes included delivery of (a) any systemic therapy; (b) any second‐line infusional therapy, following first‐line infusional therapy; and (c) any second‐line immunotherapy, following first‐line infusional therapy. Trends in care patterns associated with second‐line immunotherapy approvals in 2015 were assessed using generalized additive models. Sociodemographic and clinical predictors of treatment were explored using logistic regression.

**Results:**

Among 10 303 patients, 5173 (50.2%) received first‐line systemic therapy, with little change between the years 2012 (47.5%) and 2015 (50.3%). Among 3943 patients completing first‐line infusional therapy, the proportion starting second‐line infusional treatment remained stable from 2012 (30.5%) through 2014 (32.9%), before increasing in 2015 (42.4%) concurrent with second‐line immunotherapy approvals. Factors associated with decreased utilization of any therapy included age, black race, Medicaid eligibility, residence in a high‐poverty area, nonadenocarcinoma histology, and comorbidity; factors associated with increased utilization of any therapy included Asian race and Hispanic ethnicity. Among patients who received first‐line infusional therapy, factors associated with decreased utilization of second‐line infusional therapy included age, Medicaid eligibility, nonadenocarcinoma histology, and comorbidity; Asian race was associated with increased utilization of second‐line infusional therapy.

**Conclusion:**

United States Food and Drug Administration (FDA) approvals of immunotherapy for the second‐line treatment of advanced NSCLC in 2015 were associated with increased rates of any second‐line treatment, but disparities based on social determinants of health persisted.

## Graphical abstract

United States Food and Drug Administration (FDA) approvals of immunotherapy for the second‐line treatment of advanced non‐small cell lung cancer in 2015 were associated with increased rates of any second‐line treatment in the Medicare population. However, disparities in immunotherapy uptake based on social determinants of health persisted, driven largely by differences in rates of any first‐line treatment.

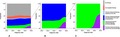

## 1 INTRODUCTION

Lung cancer is the leading cause of cancer death in the United States; most patients have distant metastatic disease at diagnosis, and over 150 000 die annually.^1^ Although palliative‐intent systemic anticancer therapy is available, estimates of the proportion of patients actually receiving such treatment on a population basis have varied from 26% to 81%.^2^, ^3^, ^4^, ^5^, ^6^ The high proportion of untreated patients likely relates to the fact that the historical backbone of systemic treatment has been cytotoxic chemotherapy. The median age at lung cancer diagnosis is 70^7^; for elderly patients with multiple comorbidities, chemotherapy too often confers only a small benefit with substantial toxicity.^8^

Fortunately, novel therapies are advancing the treatment of advanced non‐small cell lung cancer (NSCLC). Oral targeted therapy for driver mutations has improved outcomes compared with traditional chemotherapy.^9^, ^10^, ^11^, ^12^, ^13^, ^14^, ^15^, ^16^, ^17^, ^18^, ^19^, ^20^, ^21^, ^22^, ^23^, ^24^, ^25^ However, most patients do not have tumors with targetable mutations.^26^ In contrast, immunotherapy with immune checkpoint inhibitors (ICIs) has rapidly been incorporated into the standard of care for nearly all patients without targetable mutations since 2015, initially based on clinical trials demonstrating improved overall survival compared with second‐line chemotherapy.^27^, ^28^, ^29^, ^30^, ^31^

Rates of high‐grade adverse events are generally lower for immunotherapy than for cytotoxic chemotherapy for lung cancer.^27^, ^28^ Immunotherapy may therefore be an option for patients who might otherwise forego systemic therapy. This dynamic—introduction of a widely available, entirely new class of therapy—could increase the proportion of patients in particular contexts who receive treatment at all, potentially constituting a mechanism of population‐level impact that would not be evident in clinical trials. Under 5% of adults with cancer enroll on clinical trials, and elderly patients with multiple comorbidities are especially underrepresented.^32^ Real‐world evidence^33^ is therefore necessary to understand the impact of immunotherapy on treatment patterns and outcomes for patients with lung cancer on a population basis.

The objectives of this study, therefore, were to describe trends in first‐ and second‐line systemic therapy delivery with the advent of the immunotherapy era among Medicare‐insured older patients with stage IV NSCLC in the United States.

## 2 METHODS

### 2.1 Study design

This was a retrospective cohort study of older patients with stage IV non‐small cell lung cancer. The data source was SEER‐Medicare, which contains linked cancer registry and administrative data^34^ and is available from the National Cancer Institute (http://healthcaredelivery.cancer.gov/seermedicare/). The analysis was declared exempt from review by our Institutional Review Board.

### 2.2 Study subjects

Patients with a first primary lung cancer with distant metastasis at diagnosis were identified using cancer registry data. Diagnosis at age 66 or older and at least one year of Medicare parts A (inpatient), B (outpatient), and D (prescription drug) fee‐for‐service coverage before the registry diagnosis date was required, in order to estimate comorbidity antecedent to the diagnosis of cancer. Additional requirements included continuous fee‐for‐service Medicare A, B, and D coverage for at least one year after diagnosis or until death. Treatment pattern analyses were conducted within three groups: (a) all patients; (b) the subgroup that received any first‐line infusional therapy and discontinued it within one year; and (c) the subgroup that received both first‐ and second‐line infusional therapy within one year. In this analysis, the term “systemic therapy” refers to any medication used to treat cancer, and the term “immunotherapy” refers to immune checkpoint inhibitors. The term “infusional therapy” refers to any systemic therapy which would be billed under Medicare part B, including cytotoxic chemotherapy, immune checkpoint inhibitors, or other monoclonal antibodies such as bevacizumab. Oral cytotoxic chemotherapies that have an infusional equivalent, such as topotecan, would be included in this “infusional” category, although these oral cytotoxic agents do not play a major role in the treatment of NSCLC.^35^ The term “targeted therapy” refers to any oral tyrosine or serine‐threonine kinase inhibitor, which would be billed under Medicare part D.

### 2.3 Outcome variables

Treatment patterns were assessed over the 12 months following cancer diagnosis. This time window was chosen to facilitate assessments of secular trends in second‐line treatment rates over time, by maintaining the diagnosis date as the index event. Alternative methods, such as defining the index event as the completion of first‐line therapy or employing a time‐to‐event framework to enable assessment of treatments beginning more than a year after diagnosis, could have introduced confounding by clinical status at the completion of first‐line therapy and made it difficult to assess secular trends; using those methods, patients still being followed later into the immunotherapy era would have been those with the best outcomes on first‐line therapy.

The primary outcome variables of interest, therefore, were the rate of (a) any first‐line systemic therapy within one year among all patients; (b) any second‐line infusional therapy, among patients who received first‐line infusional therapy and discontinued it within one year; and (c) and any second‐line immunotherapy within one year among patients who received both first‐ and second‐line infusional therapy. Treatment was defined based on claims for drugs used for the treatment of non‐small cell lung cancer (Table S1). Sensitivity analyses were performed to evaluate (a) the impact on the calculated treated proportion of including claims for delivery of cancer treatment without drug claims, among all patients; and (b) the impact on the calculated treatment proportions of assuming that any nonspecific drug claims (HCPCS codes J3490 or J9999, which may have been submitted by providers before specific immunotherapy drug codes were widely used) represented claims for immunotherapy.

### 2.4 Line of therapy definition

In this analysis, a line of therapy was defined as any group of anti‐cancer drugs, each of which was initiated within three weeks of the first drug in the group; and ending with the last date of administration of the last drug in the group prior to either the end of follow‐up or initiation of a new drug not in the original group. Gaps in treatment alone were not considered to advance the line of therapy. Lines of therapy could contain one or more anticancer drugs. Carboplatin and cisplatin were considered equivalent for defining lines of therapy.

### 2.5 Independent variables

Independent variables included date of diagnosis; age at cancer diagnosis, divided into categories (age 66‐70, 71‐75, 76‐80, 81‐85, or 86+); sex; race, as defined by SEER; Hispanic ethnicity; urban/rural status of county of residence^36^; the ecological poverty rate among individuals aging 65‐74 who resided in each patient's census tract, divided into quintiles; the ecological rate of college education in each patient's census tract, divided into quintiles; comorbidity as calculated using the NCI comorbidity index based on claims ranging from one year before diagnosis to three months before diagnosis^37^; and any dual Medicaid enrollment^38^ in the year prior to diagnosis. For the outcome of any second‐line immunotherapy among patients who received both first‐ and second‐line infusional therapy, date of diagnosis was excluded as an independent variable from the multivariable model to preserve model stability, given the strong collinearity between diagnosis date and immunotherapy treatment rate.

### 2.6 Statistical analysis

Treatment pattern trends were analyzed using generalized additive models in which diagnosis date was the independent variable.^35^, ^39^ Predictors of each treatment pattern outcome were explored using multivariable logistic regression. Analyses were performed using SAS, version 9.4, and R, version 3.5.1.

## 3 RESULTS

### 3.1 Any systemic therapy

**Figure 1:**
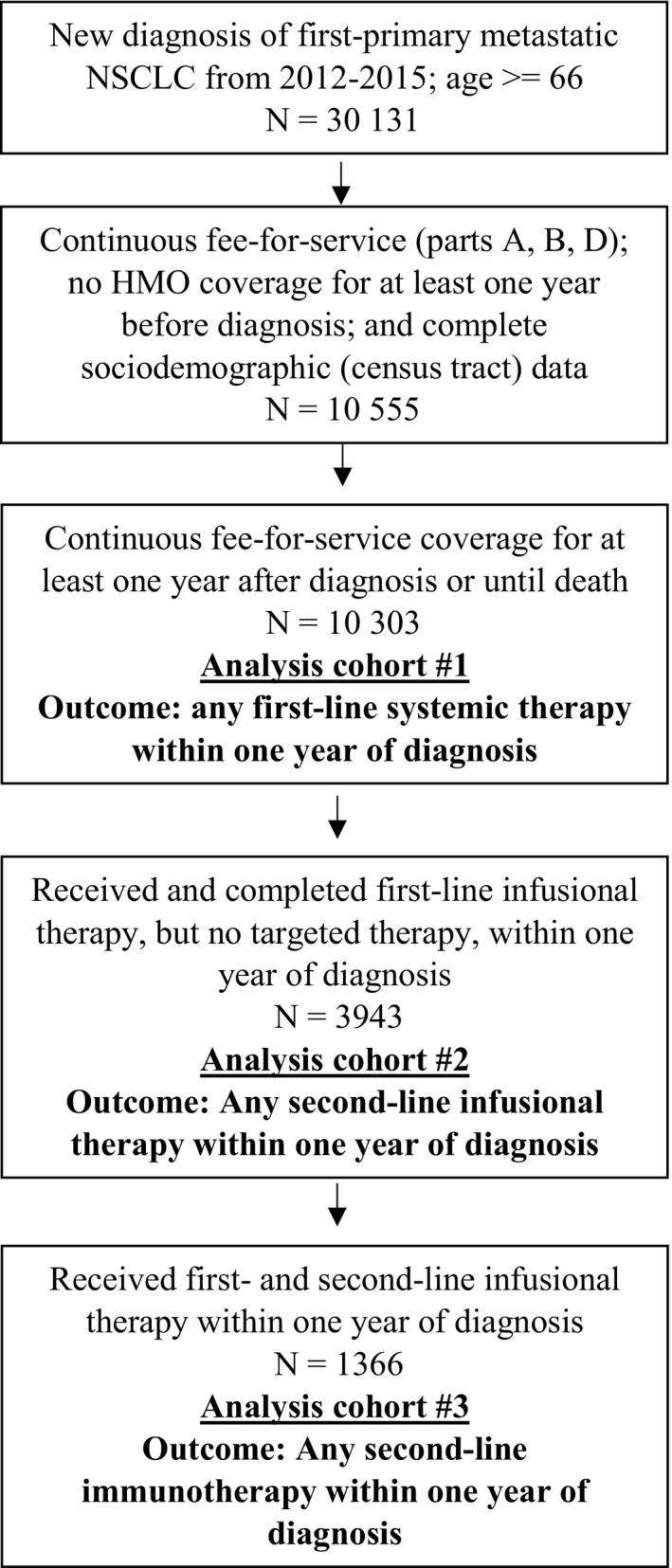
Cohort definitions

**Figure 2:**
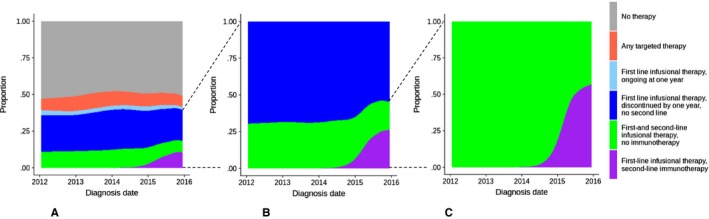
Treatment patterns for stage IV NSCLC diagnosed 2012‐2015 within one year of diagnosis (N = 10 303). A, Treatment patterns among all patients (N = 10 303). B, Treatment patterns among patients who completed first‐line infusional therapy (N = 3943). C, Treatment patterns among patients who received first‐ and second‐line infusional therapy (N = 1366). NSCLC, non‐small cell lung cancer

Cohort derivation is detailed in Figure 1; patient characteristics are provided in Table 1. Of 10 303 patients who met the entry criteria, 5173 (50.2%) had claims for any first‐line systemic therapy within one year of diagnosis, including 4246 (41.2%) with claims for any first‐line cytotoxic chemotherapy, 712 (6.9%) with claims for any first‐line targeted therapy, and 681 (6.7%) with any first‐line nonspecific drug claims. Only 16 (0.2%) had claims for any first‐line immunotherapy, as expected, since all cohort patients were diagnosed before first‐line immunotherapy was approved for NSCLC in 2016.^40^ Among all 10 303 patients, 7619 (73.9%) died within one year of diagnosis, including 2924 (56.5%) of the 5173 patients who received first‐line systemic therapy and 4695 (91.5%) of the 5130 who did not. There was no clear trend in the proportion of patients receiving any systemic therapy from 2012 to 2015 (Figure 2). In a sensitivity analysis, allowing nonspecific drug claims to define first‐line therapy in the absence of any specific drug claims increased the proportion of treated patients only slightly, from 50.2% to 53.3%.

**Table 1:** Cohort characteristics and treatment patterns

	All stage IV NSCLC patients		Stage IV NSCLC patients who received and completed first‐line infusional therapy^a^ within one year of diagnosis		Stage IV NSCLC patients who received first‐ and second‐line infusional therapy^a^ within one year of diagnosis	
	N (%)	% receiving any systemic therapy	N (%)	% receiving second‐line infusional therapy^a^	N (%)	% receiving second‐line immunotherapy
All patients	10 303 (100)	50.2	3943 (100)	34.6	1366 (100)	16.2
Diagnosis year						
2012	2431 (24)	47.5	859 (22)	30.5	262 (19)	0.0
2013	2536 (25)	50.9	962 (24)	31.7	305 (22)	0.0
2014	2647 (26)	51.9	1053 (27)	32.9	346 (25)	3.8
2015	2689 (26)	50.3	1069 (27)	42.4	453 (33)	45.9
Age						
66‐70	2631 (26)	58.8	1224 (31)	39.3	481 (35)	16.6
71‐75	2888 (28)	57.2	1327 (34)	34.4	457 (34)	13.6
76‐80	2187 (21)	50.6	839 (21)	33.6	282 (21)	18.1
81‐85	1606 (16)	37.9	413 (11)	26.6	110 (8)	18.2
86+	991 (10)	26.2	140 (4)	25.7	36 (3)	f
Sex						
Female	5282 (51)	50.9	1857 (47)	36.6	680 (50)	16.9
Male	5021 (49)	49.6	2086 (53)	32.9	686 (50)	15.5
Race^b^						
White	8526 (83)	50.5	3399 (86)	34.5	1173 (86)	15.9
Black	980 (10)	41.2	328 (8)	31.4	103 (8)	18.4
Asian/other/unknown	797 (8)	58.0	216 (6)	41.7	90 (7)	17.8
Ethnicity^b^						
Non‐Hispanic	9738 (95)	50.2	3741 (95)	34.6	1295 (95)	16.4
Hispanic	565 (6)	49.6	202 (5)	35.1	71 (5)	f
Medicaid enrollment^c^						
No	7098 (69)	54.0	2998 (76)	35.8	1072 (79)	17.1
Yes	3205 (31)	41.7	945 (24)	31.1	294 (22)	12.9
Area‐level college education (quintile)						
1	2002 (19)	49.0	738 (19)	37.7	278 (20)	15.8
2	2082 (20)	50.0	775 (20)	33.7	261 (19)	17.6
3	2025 (20)	50.8	820 (21)	32.4	266 (20)	14.9
4	2122 (21)	49.7	777 (20)	34.5	268 (20)	14.9
5	2072 (20)	51.6	833 (21)	35.2	293 (21)	17.1
Area‐level poverty rate (quintile)^d^						
1	2125 (21)	53.6	855 (22)	36.4	311 (23)	17.7
2	2083 (20)	53.3	826 (21)	38.1	315 (23)	14.9
3	2057 (20)	51.5	805 (20)	32.5	262 (19)	16.0
4	2039 (20)	47.3	749 (19)	32.2	241 (18)	16.2
5	1999 (19)	44.9	708 (18)	33.5	237 (17)	16.0
Urban‐rural status^e^						
Large metro	5234 (51)	51.4	1950 (50)	36.9	720 (53)	14.9
Metro	3073 (30)	49.3	1189 (30)	33.1	393 (29)	19.6
Urban	642 (6)	50.2	260 (7)	33.1	86 (6)	16.3
Less urban	1079 (11)	47.0	427 (11)	29.7	127 (9)	13.4
Rural	275 (3)	49.8	117 (3)	34.2	40 (3)	f
NCI comorbidity score^g^						
0	4290 (42)	55.8	1747 (44)	38.7	676 (50)	17.5
1	2767 (27)	52.4	1114 (28)	32.1	358 (26)	15.1
2+	3246 (32)	40.9	1082 (27)	30.7	332 (24)	14.8
NSCLC histology						
Adenocarcinoma	6317 (61)	52.8	2284 (58)	37.0	845 (62)	14.9
Large cell carcinoma	228 (2)	47.4	98 (3)	30.6	30 (2)	f
Not specified	907 (9)	41.0	316 (8)	28.8	91 (7)	17.6
Squamous cell	2851 (28)	47.5	1245 (32)	32.1	400 (29)	19.2

Abbreviation: NSCLC, non‐small cell lung cancer.

a Infusional therapy refers to any cytotoxic chemotherapy, immune checkpoint inhibitor, or other monoclonal antibody.

b Race and ethnicity as reported by SEER.

c Medicaid dual eligibility defined using any state buy‐in^38^ within the 12 mo prior to diagnosis according to Medicare enrollment data.

d Ecological poverty rate among people aging 65‐74 in each patient's census tract of residence.

e Urban‐rural code as defined by the US Department of Agriculture and categorized by the NCI.^36^

f Redacted per National Cancer Institute requirements to preserve patient confidentiality.

g Comorbidity score per the National Cancer Institute modification of the Charlson comorbidity index.^47^

When treatment patterns over the entire first year after diagnosis were analyzed, 944 patients (9.2% of the full cohort, or 18.2% of treated patients) received targeted therapy in the first line or thereafter. Of the remaining 4229 patients treated with infusional therapy only, just 286 (6.8%) remained on first‐line therapy by one year after diagnosis; with censoring at the one‐year timepoint, the median time on first‐line treatment among patients receiving chemotherapy was only 2.0 months. The remaining 3943 patients constituted the cohort for subsequent second‐line infusional therapy analyses. Of the 16 patients who received first‐line immunotherapy, some also received targeted therapy, and the remainder were analyzed together with the patients who received first‐line infusional therapy (data not shown to preserve confidentiality as per National Cancer Institute requirements). In a second sensitivity analysis, imposing an assumption that non‐specific infusional systemic therapy claims (HCPCS codes J3490 or J9999) represented claims for immunotherapy had little impact on overall treatment pattern trends (Figure S1).

**Figure 3:**
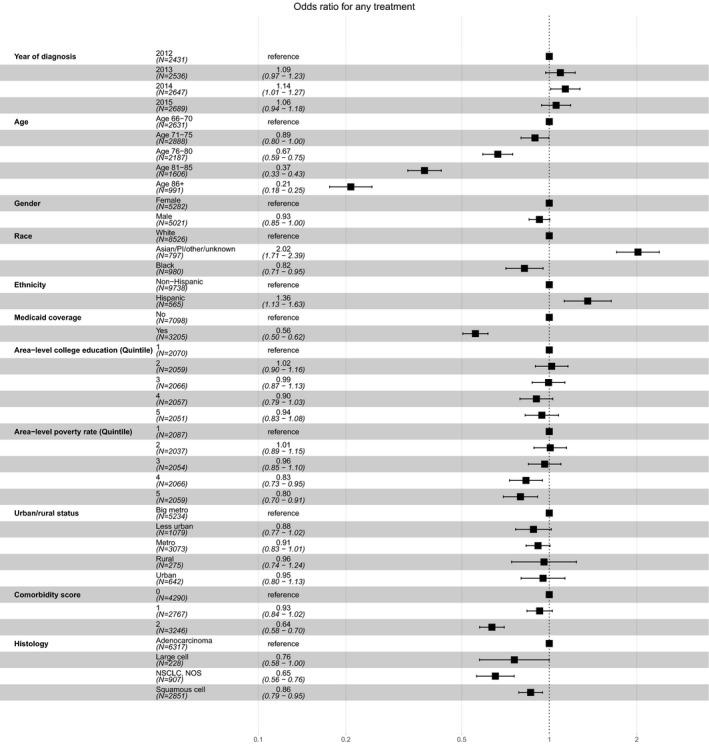
Sociodemographic and clinical predictors of any systemic therapy for stage IV NSCLC within one year of diagnosis (n = 10 303). NSCLC, non‐small cell lung cancer

Patients who were older, Black, or had at least two comorbidities, nonadenocarcinoma histology, dual Medicaid coverage, or lived in high‐poverty census tracts were less likely to receive any systemic therapy; patients living in rural areas were no less likely to receive systemic therapy. Patients of Asian/Pacific Islander/Other/unknown descent or Hispanic ethnicity were more likely to receive systemic therapy (Table 1; Figure 3).

### 3.2 Second‐line infusional therapy

**Figure 4:**
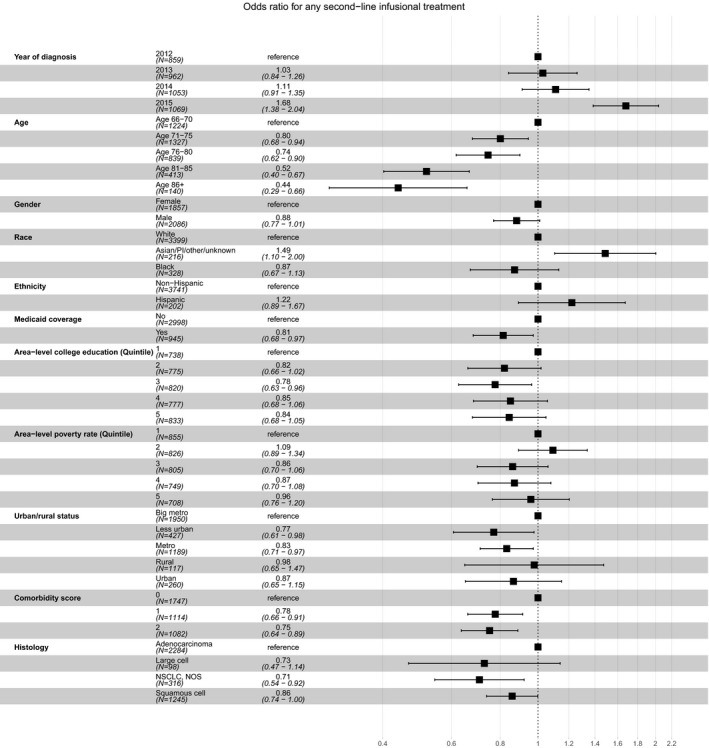
Sociodemographic and clinical predictors of second‐line infusional therapy among patients completing first‐line infusional therapy (n = 3943)

Among the 3943 patients who received first‐line infusional treatment but discontinued it by one year after diagnosis, and who did not receive targeted therapy during the year after diagnosis, 1366 (34.6%) also initiated second‐line infusional treatment within that year. The rate of second‐line infusional treatment was 30.5% in 2012, 31.7% in 2013, 32.9% in 2014, and 42.4% in 2015 (Figure 2); *P* < .001 by the chi‐square test for the comparison of second‐line infusional treatment rates among patients diagnosed in 2015 versus 2014. Patients who were older, dually eligible for Medicaid, had nonadenocarcinoma histology, or had more comorbidities were less likely to proceed from first‐line to second‐line infusional therapy; patients of Asian/Pacific Islander/Other/unknown descent were more likely to do so (Table 1; Figure 4).

### 3.3 Second‐line immunotherapy

**Figure 5:**
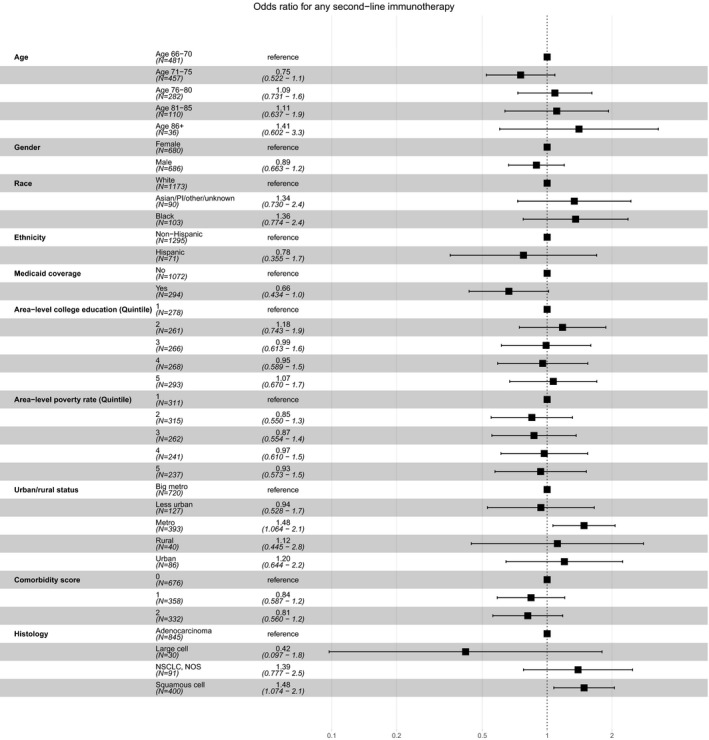
Sociodemographic and clinical predictors of second‐line immunotherapy among patients completing first‐ and second‐line infusional therapy (n = 1366)

Among the 1366 patients who received first‐ and second‐line infusional therapy, 221 (16.2%) overall received second‐line immunotherapy. As expected, no patients diagnosed in 2012 or 2013 received second‐line immunotherapy within one year. Among 346 patients in this subgroup diagnosed in 2014, 13 (3.8%) received second‐line immunotherapy within one year; among 453 patients in this subgroup diagnosed in 2015, 208 (45.9%) received second‐line immunotherapy within one year. Proceeding to second‐line immunotherapy was slightly more common among patients living in “metropolitan” (versus “large metropolitan” areas) and those who had squamous cell histology (Table 1; Figure 5).

## 4 DISCUSSION

In this analysis of treatment patterns among older patients with metastatic NSCLC diagnosed immediately before and after the first approvals of immunotherapy for NSCLC in the second‐line setting, we found persistently low rates of any systemic treatment, with disparities by race and socioeconomic status, consistent with prior studies predating the immunotherapy era.^2^, ^3^, ^4^, ^5^, ^6^ The overall rate of systemic therapy delivery did not change substantially with the availability of second‐line immunotherapy subsequent to its approval by the United States Food and Drug Administration (FDA) for the second‐line treatment of squamous NSCLC in March 2015 and nonsquamous NSCLC in October 2015. However, among patients who received first‐line infusional therapy, the use of immunotherapy for second‐line treatment increased rapidly following FDA approval and was associated with a modest increase in the rate of any second‐line therapy.

Overall, the uptake of immunotherapy among older adults on a population basis in the United States appears to have been rapid. Within one year of initial approval, most patients initiating second‐line treatment were receiving immunotherapy. However, despite this rapid uptake, less than 10% of all patients diagnosed in 2015 received immunotherapy within a year of diagnosis. This was largely attributable to the high proportion of patients who received no systemic therapy at all, since few patients remained on first‐line therapy for a full year (Figure 2). This finding has important implications for future research. Importantly, the indications for the use of immunotherapy expanded to include use in the first‐line treatment of advanced NSCLC without targetable mutations in October 2016.^35^ Given the size of the previously untreated population, assessment of the effectiveness and value of first‐line immunotherapy will need to measure the extent to which immunotherapy expands the population of treated patients. In other words, relevant effectiveness analysis will therefore compare not just immunotherapy versus traditional treatments, but immunotherapy versus no treatment at all.

This study has several strengths. It is based on a cohort of older patients that is representative of the population of SEER registry sites. This facilitates unbiased examination of rates of any treatment, including first‐line treatment, which is less feasible in cohorts drawn from oncology practices,^41^, ^42^ consisting of patients more likely to be treated. This distinction is especially important in light of the poor survival in our population‐based cohort. Still, the results confirm a recent report from oncology practices that second‐line treatment for NSCLC became more common in the immunotherapy era.^43^ In our analysis, it did not appear that the increase in second‐line treatment within one year of diagnosis related to any substantial decrease in the proportion of patients remaining on first‐line therapy for a full year, which was an uncommon outcome in this population across years of diagnosis (Figure 2). Limitations include the lag between clinical events and data availability in SEER‐Medicare, such that it is not yet possible to assess the impact of first‐line immunotherapy approvals^40^, ^44^, ^45^ on population treatment patterns. Further research will be necessary to evaluate this impact when updated data become available. Finally, this analysis was restricted to patients with stage IV (de novo metastatic) disease; algorithms for defining recurrent disease using claims data^46^ are challenging to apply to the question of rates of any treatment, since treatment claims are an important component of identifying recurrence.

In conclusion, a large proportion of older patients with metastatic NSCLC diagnosed from 2012‐2015 did not receive systemic therapy, and socioeconomic disparities in these rates persisted. The advent of immunotherapy was associated with an increase in the proportion of patients receiving second‐line treatment after discontinuing first‐line chemotherapy. Assessment of the effectiveness and value of immunotherapy should include measurement of its impact on the proportion of patients receiving any treatment.

## CONFLICTS OF INTEREST

Dr Kehl reports serving as a consultant to Aetion, Inc Dr Schrag reports receiving research funding from Pfizer and the American Association of Cancer Research, and serving as an editor and receiving personal fees from the Journal of the American Medical Association. Dr Hassett has no relevant conflicts of interest to disclose.

## AUTHOR CONTRIBUTIONS

Kenneth L. Kehl: conceptualization, data curation, formal analysis, funding acquisition, investigation, methodology, writing—original draft, writing—review, and editing. Michael J. Hassett: conceptualization, funding acquisition, investigation, methodology, writing—review, and editing. Deborah Schrag: conceptualization, investigation, methodology, writing—review, and editing.

## Data Availability

The data source was SEER‐Medicare, which contains linked cancer registry and administrative data^34^ and is available from the National Cancer Institute (http://healthcaredelivery.cancer.gov/seermedicare/).
