# Supplementary material for: Patterns of care for older patients with stage IV non‐small cell lung cancer in the immunotherapy era
Source: Cancer Med. 2020 Jan 27;9(6):2019–29. doi: 10.1002/cam4.2854 (PMC7064091; doi:10.1002/cam4.2854)
Supplement: Supplementary file 1 [file CAM4-9-2019-s001.docx]

Supplemental Figure 1: Sensitivity analysis of treatment patterns among patients diagnosed with stage IV NSCLC 2012-2015, treating nonspecific drug claims (HCPCS codes J3490 or J9999) as claims for immunotherapy


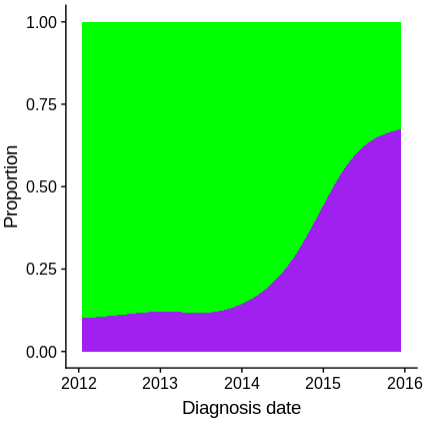
**
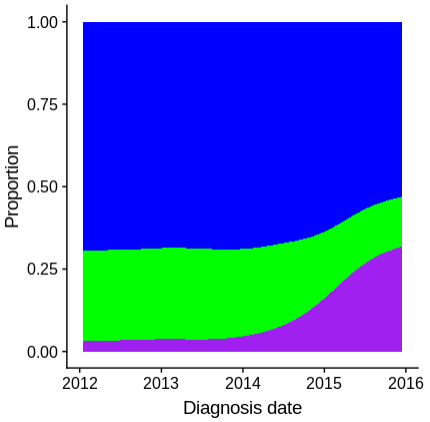

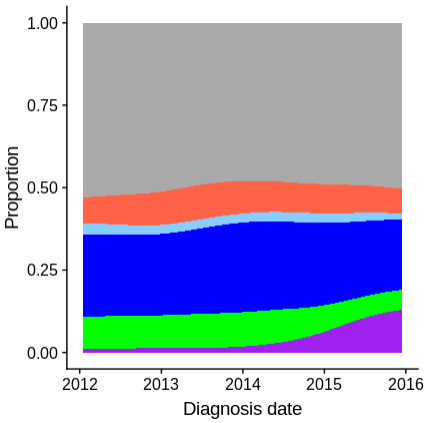

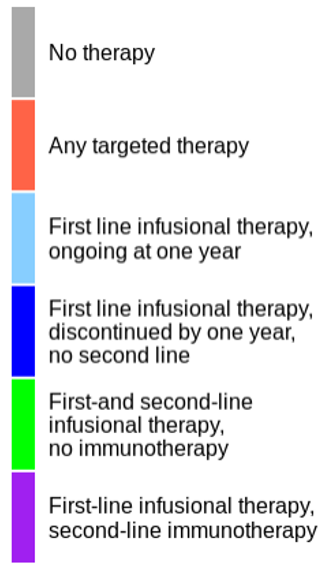
**

Figure 2A: Treatment patterns among all patients (N=10,303)

Figure 2B: Treatment patterns among patients who completed first-line infusional therapy (N=3,943)

Figure 2C: Treatment patterns among patients who received first- and second-line infusional therapy

(N=1,366)

Supplemental Table 1: Specific drugs assessed for the treatment of stage IV NSCLC*

| “Infusional” therapy: Immunotherapy | “Infusional” therapy: Cytotoxic | “Infusional” therapy: Other | Targeted therapy |
| --- | --- | --- | --- |
| Atezolizumab | Capecitabine | Bevacizumab | Afatinib |
| Avelumab | Carboplatin | Ramucirumab | Alectinib |
| Durvalumab | Cisplatin | Necitumumab | Brigatinib |
| Nivolumab | Cyclophosphamide |  | Ceritinib |
| Pembrolizumab | Docetaxel |  | Crizotinib |
|  | Doxorubicin |  | Dabrafenib |
|  | Epirubicin |  | Erlotinib |
|  | Eribulin |  | Gefitinib |
|  | Etoposide |  | Osimertinib |
|  | Fluorouracil |  | Trametinib |
|  | Gemcitabine |  | Vemurafenib |
|  | Mechlorethamine |  |  |
|  | Paclitaxel |  |  |
|  | Pemetrexed |  |  |
|  | Nap-paclitaxel |  |  |
|  | Thiotepa |  |  |
|  | Vinblastine |  |  |
|  | Vincristine |  |  |
|  | Vinorelbine |  |  |
|  | Topotecan |  |  |

* Consists of drug claims assessed for this analysis; not all drugs were administered to any cohort patients or were commonly used/approved to treat NSCLC during the time of this study.
